# Apples and Apple By-Products: Antioxidant Properties and Food Applications

**DOI:** 10.3390/antiox12071456

**Published:** 2023-07-19

**Authors:** Umme Asma, Ksenia Morozova, Giovanna Ferrentino, Matteo Scampicchio

**Affiliations:** Faculty of Agricultural, Environmental and Food Sciences, Free University of Bozen-Bolzano, Piazza Università 1, 39100 Bolzano, Italy; asma@unibz.it (U.A.); ksenia.morozova@unibz.it (K.M.); matteo.scampicchio@unibz.it (M.S.)

**Keywords:** natural antioxidants, food additives, phenolic content, extraction

## Abstract

In recent years, there has been a growing interest in utilizing natural antioxidants as alternatives to synthetic additives in food products. Apples and apple by-products have gained attention as a potential source of natural antioxidants due to their rich phenolic content. However, the extraction techniques applied for the recovery of phenolic compounds need to be chosen carefully. Studies show that ultrasound-assisted extraction is the most promising technique. High yields of phenolic compounds with antioxidant properties have been obtained by applying ultrasound on both apples and their by-products. Promising results have also been reported for green technologies such as supercritical fluid extraction, especially when a co-solvent is used. Once extracted, recent studies also indicate the feasibility of using these compounds in food products and packaging materials. The present review aims to provide a comprehensive overview of the antioxidant properties of apples and apple by-products, their extraction techniques, and potential applications in food products because of their antioxidant or nutritional properties. The findings reported here highlight the proper utilization of apples and their by-products in food to reduce the detrimental effect on the environment and provide a positive impact on the economy.

## 1. Introduction

Natural antioxidants derived from fruits and vegetables have gained interest as important food additives and nutraceutical supplements. The number of publications related to antioxidants and their applications has significantly increased in the last few years [1,2,3,4,5]. The purpose of using antioxidants in a food product is to delay the oxidation reaction by inhibiting the formation of free radicals or by interrupting the propagation of free radicals [6,7,8]. In general, many plant tissues develop antioxidant compounds under constant oxidative stress to protect themselves from free radicals, reactive oxygen species, and pro-oxidants generated both exogenously (heat and light) and endogenously (H_2_O_2_ and transition metals). These antioxidant species are known as natural antioxidants. Some common examples of natural antioxidants are flavonoids, phenolic acids, carotenoids, and vitamins (ascorbic acid, beta-carotene, tocopherols). They are efficient in scavenging free radicals, chelating pro-oxidant metal ions, or acting as reducing agents [1,9,10].

**Figure 1:**
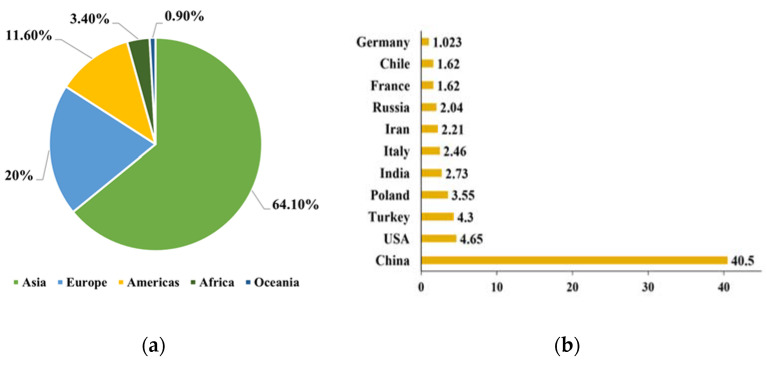
The worldwide production of apples from 2012 to 2020 (**a**) and top 11 producers of apples in 2020 (**b**) according to data provided by the Food and Agriculture Organization of the United Nations.

Apples (Malus domestica) are the most consumed fruit, and they contain various bioactive compounds, including pectins, dietary fibers, vitamins, oligosaccharides, triterpenic acids, and phenolic compounds such as flavonols, dihydrochalcones, anthocyanidins, hydroxycinnamic acids, and hydroxybenzoic acids. Because of the higher content of these phenolic compounds (>20 mmol TE/kg), apples are well known for their high antioxidant properties [11,12,13,14]. According to the Food and Agriculture Organization of the United Nations, more than 86 million tons of apples were produced globally in 2020. The global production of apples from 2012 to 2020 is reported in Figure 1A. Based on these data, Asia stands as the largest producer (64.10%), followed by Europe providing 20% of total global production. The Americas come in third place, contributing 11.6% of apples in the last decade. The main apple producers in Europe are Poland, France, and Italy with more than 7.6 million tons of apples in 2020 as shown in Figure 1B.

Generally, apples are consumed as fresh fruit. In 2019, more than 57 million tons of apple were consumed globally [15]. However, fresh apples deteriorate and are lost prior to consumption because of their short shelf life and the presence of spoilage pathogens. The use of preservatives, manufacturing and cooking processes including pasteurization, and the addition of some additives can reduce the spoilage of fresh fruits.

Apples are also widely consumed in the form of processed food products. Typical examples of processed products from apples include juices, sauces, jams, apple pies, apple cider vinegar, wine, and apple snacks (chips). Among all processed apple products, apple juice is the most produced product (65%) [4,16]. 

**Figure 2:**
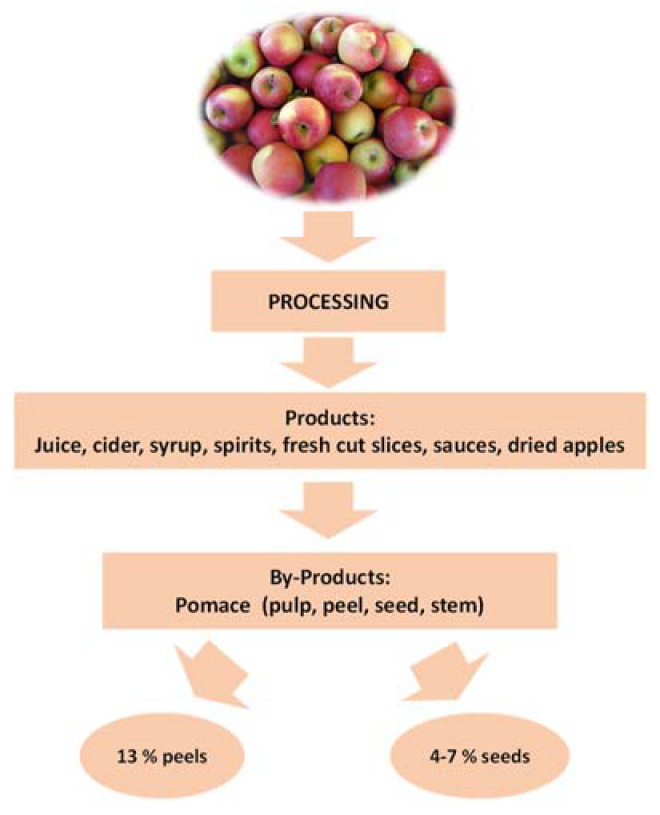
Apple by-product distribution.

During the processing of apples, a large amount of by-products is produced. This by-product is called pomace and is constituted by a mixture of peel, core, seed, calyx, stem, and soft tissue. In the following Figure 2, the distribution of apple by-products is reported.

About 13% of the weight of the fresh apple fruit is constituted by the peels, which are obtained from fresh-cut fruit and apple pie and sauce manufacturing [16]. The other by-product from the apple pomace is represented by the seeds. They are obtained by sieving and separation from apple pomace in a mass fraction ranging from 4 to 7%. Apple seeds contain amygdalin, a cyanogenic glycoside. Its degradation induced by β-glucosidase, naturally present in the human intestine, can lead to the formation of cyanide causing severe toxicity in humans [16].

Apple by-products are mainly considered as waste, with processing companies facing problems with discarding them in a sustainable way. Most of the time, apple pomace is used as fertilizer, for animal fodder, or as a substrate for aerobic fermentation [17,18]. However, the disposal of apple by-products as waste causes huge losses [19]. 

Several studies have shown that apple by-products possess high biological activities due to the high content of phenolic compounds, vitamins, and carotenoids [20]. Therefore, apples and apple by-products are potential sources from which to extract natural antioxidants to be used in the food, cosmetic, and nutraceutical industries. 

In this review, the antioxidant properties of apples and apple by-products are explained, focusing on their phenolic content, extraction techniques, and potential applications in the food industry.

## 2. Antioxidant Compounds Present in Apples and Apple By-Products

Apples have gained special attention due to their chemical composition, especially for their antioxidant characteristics. Among different groups of natural antioxidants, phenolic compounds are the main constituents responsible for the antioxidant properties of apples [1,15,21]. In the following section, the main groups (with their subgroups) of antioxidant compounds are described. 

### 2.1. Vitamins

Apples are a rich source of vitamins, especially vitamins C and E. With regard to vitamin C content, apples are placed as the second highest ranked fruit after cranberries [21]. The concentration of vitamin C ranges from 2 to 35 mg/100 g based on the apple variety, and it is found in two forms in apples, namely ascorbic acid and its oxidized form, dehydroascorbic acid [15,22]. It has been reported that vitamin C has antioxidant properties with free radical-scavenging activity of EC50 = 0.35 (here EC50 is the concentration required to obtain a 50% antioxidant effect). On the other hand, vitamin E is mainly present in the apple seeds. As a result, apple pomace is a rich source of vitamin E, and it has been found that the concentration of vitamin E in apple pomace is 5.5 mg/100 g with a free radical-scavenging activity of EC50 = 0.30. Other vitamins found in apples are vitamin B12 and vitamin D, but they are present in trace amounts [21].

### 2.2. Phenolic Compounds

**Figure 3:**
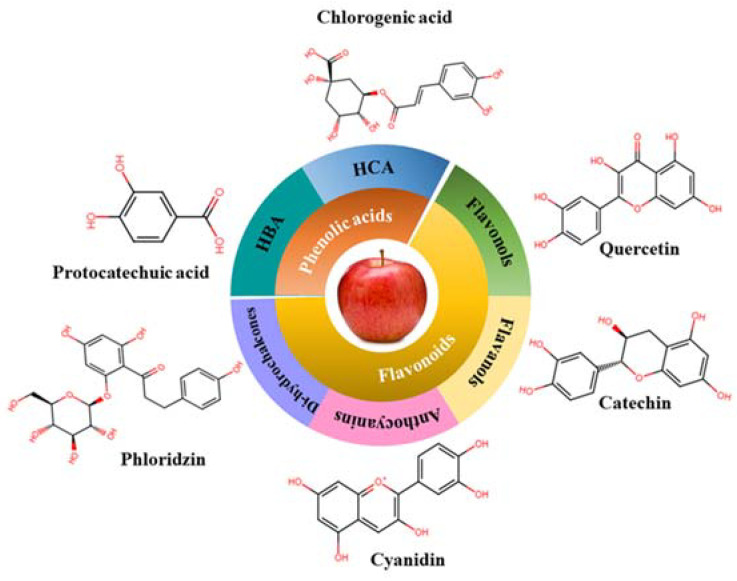
Apple and apple by-product phenolic compounds.

Phenolic compounds are one of the largest classes of plant secondary metabolites with biological functions in humans. They contain one or more aromatic rings in their molecular structures with one or more hydroxyl groups, which are responsible for exhibiting biological functions. To date, more than 60 phenolic compounds have been found in apples [13,23], which are mainly constituted by phenolic acids and flavonoids (Figure 3). 

#### 2.2.1. Phenolic Acids

Phenolic acids are the major components belonging to the non-flavonoid group of phenolic compounds. They are aromatic acids with a phenolic ring and an organic carboxylic acid (C6-C1 skeleton). They are also known as phenol carboxylic acids. According to their classification, there are two main types: hydroxybenzoic acids (C6-C1) and hydroxycinnamic acids (C6-C3) [24]. 

Hydroxybenzoic acids are derivatives of benzoic acid, found in fruits mostly in the conjugated form (esters or glycosides) but can also be present in the free form. Generally, they are bound to components of cell walls (such as cellulose or lignin or even forming protein complexes) that can be connected to sugars or organic acids [25]. Examples of hydroxybenzoic acids present in apples include gallic acid, protocatechuic acid, vanillic acid, and syringic acid [13,23,26,27]. On the other hand, hydroxycinnamic acids are rarely available in free form and are mainly present in conjugated forms (glycosylated or esters). The most reported hydroxycinnamic acids found in apples are quinic and caffeic acid, and the estimated range is 4 to 18% of total phenolic compounds based on apple varieties. Furthermore, 5′-caffeoylquinic or chlorogenic acid, p-coumaroylquinic, and p-coumaric are also present in apple. They are more abundant in apple peel in comparison to apple flesh [25,26,28].

#### 2.2.2. Flavonoids

Apples are enriched with flavonoids. Among eight subclasses of flavonoid, apples contain mainly four subclasses, namely flavonols (71–90%), flavanols-3-ols (1–11%), anthocyanins (1–3%), and chalcones/dihydrochalcones (2–6%). The most abundant flavonols found in apples are quercetin glycosides (with quercetin 3-glycoside), whereas catechins, epicatechins, and procyanidin B2 are the major compounds belonging to flavan-3-ols (they are mainly present in apple peel and pulp) [27,29]. The third subgroup “anthocyanins” includes cyanidin 3-galactoside, which is most abundant in red apple peel as it is responsible for the red color. Examples of phenolic compounds belonging to dihydrochalcones are phlorizin and phloretin. These compounds are more abundant in apple seeds and apple pomace and mainly found in conjugated forms (linked to the fruit sugar content, such as glucose and xyloglucan) [30,31].

## 3. Extraction of Phenolic Compounds from Apples and Apple By-Products

The yield of total phenolic content and antioxidant properties of phenolic compounds from apples and apple by-products are greatly influenced by the extraction techniques and conditions [32,33]. The main purpose of extraction is to recover all compounds present in apple, without any chemical modifications [34]. Several studies have been published demonstrating different extraction techniques and conditions to recover phenolic compounds from apples and apple by-products. In general, the extraction of phenolic compounds from apple can be achieved by using conventional or innovative extraction techniques [35,36]. 

### 3.1. Conventional Extraction

The widely reported conventional methods for the extraction of phenolic compounds from apples are maceration, Soxhlet extraction, and hydro-distillation. Conventional Soxhlet extraction is mainly used for the recovery of volatile compounds from apples, especially aroma compounds (as the recovered extracts provide the aroma closest to the fresh fruit), whereas vacuum hydro-distillation is used to extract volatile and polar components from plant matrices [37,38]. Indeed, Soxhlet extraction was found to be effective in extracting phenolic compounds such as phlorizin, epicatechin, quercetin, and phloretin from apple pomace with a yield of phenolic compounds equal to 4.13 mg/g [39]. The right choice of solvent is a crucial factor for these extraction methods. The extraction solvent should be selected based on the polarity of phenolic compounds. For phenolic compounds, ethanol, acetone, methanol or acidified methanol, water, or a combination of water with methanol/ethanol were the most recommended solvents as mentioned by several authors. Water is a suitable solvent to extract phenolic compounds from apple pomace like hydroxycinnamic acids and flavonoids (dihydrochalcones, flavanols) [40]. However, it is not the right choice for some phenolic compounds like quercetin glycosides [41]. On the other hand, pure organic solvents alone are not sufficient to extract polar phenolic compounds. For this purpose, it is important to use more polar solvents in a combination. For example, methanol is an appropriate solvent to extract chlorogenic acid and phlorizin, and the optimized conditions for extraction using methanol were 84.5% methanol for 15 min at 28 °C. However, acetone (65%) shows high selectivity for the recovery of most polyphenols with high antioxidant properties by performing a maceration for 20 min at 10 °C [42]. Sometimes, multiple-step extraction is required to extract 100% of phenolic compounds. In multi-step extraction, different types of solvents are used. According to Reis et al. [41], three-step extraction was able to recover the total phenolic content from apple pomace. The first solvent was water, which yielded 67%, and the second and third steps were performed using methanol and acetone to obtain a yield of 17% and 16%, respectively [41].

Fromm et al. [43] used a magnetic stirrer to optimize extraction from apple seeds based on time and temperature. For this purpose, the authors conducted experiments at different temperatures (0–42 °C) and extraction times (60–1440 min) using aqueous acetone (60–70% *v*/*v*), and they reported that the optimum value was at 25 °C for 60 min. Moreover, other authors have also demonstrated that the optimum temperature was 60 °C to extract phenolic compounds from apple pomace, but reduced extraction time was observed (from 60 min to 30 min) while using aqueous methanol (80%, *v*/*v*) and ethanol (50%, *v*/*v*) [30,43].

However, the major drawbacks of conventional extraction techniques are the lack of temperature control, exposure to light, longer extraction time, and the large quantity of organic solvents required, which may reduce the extraction yields and the extract concentration of target compounds. These drawbacks have led researchers to seek innovative techniques that are closer to the concept of “green” technologies [44,45]. In fact, these alternative technologies are aimed at providing safe compounds by minimizing or eliminating the drawbacks of the conventional techniques [46].

### 3.2. Innovative Extraction

The most innovative techniques are ultrasound-assisted extraction (UAE), microwave-assisted extraction (MAE), supercritical fluid extraction (SFE), pressurized liquid extraction, and pulsed electric fields (PEF) [47]. Phenolic compounds of apple can easily be extracted by microwave-assisted extraction (MAE). In MAE, microwave energy is used to heat solvents in contact with a sample to allow the analytes partitioning from the sample matrix into the solvent. In general, ethanol is used as a solvent in this method with a specific ratio of 22.9:1 (solvent: raw material). MAE showed higher efficiency at a shorter time for the extraction of phenolic compounds from apple pomace in comparison to conventional methods (Soxhlet and maceration) [48].

Ultrasound-assisted extraction (UAE) is regarded as a sustainable technology as it needs a moderate investment of solvent and energy. Furthermore, it is very cheap, easy to operate, and reproducible due to its capability to work under atmospheric pressure and at an ambient temperature. The UAE technique shows potential to increase the yield of polyphenols thanks to the enhancement of the diffusion of a solvent through the cell walls, and thus it increases the release of the cell components (nutritional components and phenolic compounds) during extraction [49,50]. It has been reported that the yield of phenolic compounds from apple pomace using UAE (even with the same extraction conditions) is 20% and 30% higher than conventional methods [50,51]. Recently, UAE combined with natural deep eutectic solvents (NADES) has been developed. It has been reported that UAE and NADES with optimized parameters showed better efficiency in terms of TPC, TFC, and radical-scavenging activity in comparison to conventional solvents such as water, ethanol, and 30% hydro-ethanolic solution. The best NADES were found to be choline chloride in combination with glycerol (1:2) and choline chloride with lactic acid (1:3). For both these two cases, values for TPC, TFC, and radical-scavenging activity by DPPH were higher in comparison to the conventional solvents [51]. The yield for apple pomace polyphenol is also higher for the UAE method in comparison to the MAE method; the values of yield were 10.2 mg/g and 3.8 mg/g, respectively [52]. The optimal conditions for the extraction process of polyphenols from apple pomace were reported by Virot as an ultrasonic power of 0.142 W/g, a temperature of 40.1 °C, and sonication time of 45 min, whereas the best yield was reported by Pingret et al. [53] at an ultrasound intensity of 0.76 W/cm^2^ at 40 °C for 40 min [53,54].

Supercritical fluid extraction is another example of a green and efficient technology. This technology is mainly used to extract essential oils from plant-based by-products. To date, several studies have been conducted to recover high-added value compounds using this technology. Carbon dioxide is the most used fluid in supercritical fluid extraction as it possesses low toxicity, low critical temperature (31.1 °C), and a good safety profile with or without ethanol (5%) as a co-solvent. The use of organic co-solvents (e.g., ethanol, methanol, acetone) is required to increase the solubility of polar polyphenols during extraction as carbon dioxide is a non-polar solvent. By changing the extraction parameters (e.g., pressure, temperature, ethanol concentration, extraction time), it is also possible to extract specific phenolic compounds precisely from a complex mixture of natural compounds. It has been demonstrated that extraction using SFE to recover phenolic compounds from freeze-dried apple pomace at 30 MPa and 45 °C for 2 h with ethanol as a co-solvent exhibited a higher antioxidant activity (5.63 ± 0.10 mg TEA/g of extract) in comparison to conventional extraction technologies such as Soxhlet with ethanol (2.05 ± 0.21 mg TEA/g of extract) and boiling water maceration (1.14 ± 0.01 mg TEA/g of extract) [39,55].

Finally, although UAE showed promising results for the extraction of polyphenols from apples and apple by-products, further research studies should be carried out to demonstrate the optimal process conditions for each extraction technique in order to recover phenolic compounds with high antioxidant activity using kinetic approaches. 

By comparing the performances of the innovative technologies, it is clear that depending on the nature of the used solvents, the extraction efficiency changes drastically. It has been demonstrated that, due to the nature of supercritical carbon dioxide, extraction of apolar compounds occurs during SFE. Moreover, although SFE is a green technique, the yield in extracting compounds from apples and apple by-products is quite low compared to UAE and MAE where organic solvents are used. To improve this aspect, the use of a co-solvent is addressed, which could tune the extraction towards the recovery of polar compounds. 

## 4. Applications of Apple Phenolic Compounds in Food Products

**Figure 4:**
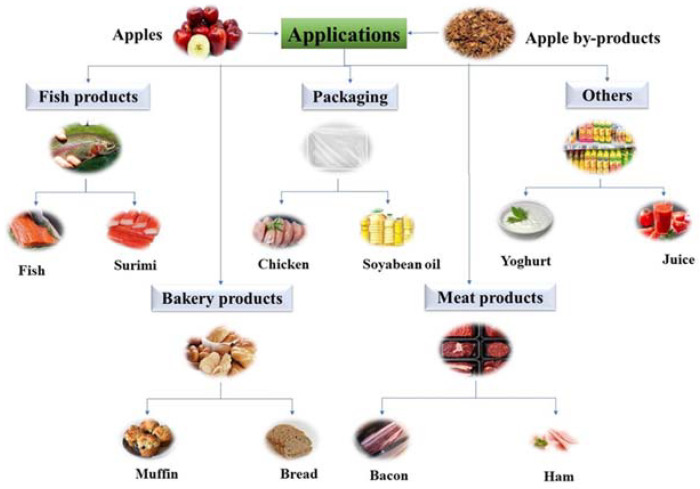
Application of apple phenolic compounds in different foods.

Nowadays, fortification of food products using apples or apple by-products has gained more attention as they are a good source of dietary fibers and bioactive compounds. These products, rich in natural antioxidants, could play an important role in replacing synthetic products [56,57]. In the following subsections, food products formulated with the incorporation of apples or by-products are described as shown in Figure 4. The applications of apples or by-products are listed in Table 1.

### 4.1. Bread and Bakery Products

There has been a growing interest in utilizing apple and apple by-product powder in bread and bakery products (as baked goods are suitable foods to be fortified). The addition of dried apple powder (5% and 10%) increased the antioxidant capacity of wheat bread by up to 38.5% and 61.9% when compared with control bread. Overall, the sensory properties were acceptable regardless of the control samples [56]. Furthermore, recent studies have demonstrated that the total polyphenol content and antioxidant potential of wheat bread increased when formulated with defatted 5% and 20% apple seed powder of different varieties (Golden Delicious, Idared, and Šumatovka). The results showed that the total polyphenol content was 1.7 and 2.9 times higher, with 1.1 and 2.1 times higher antioxidant capacity in comparison to the control bread [58]. In addition, the antioxidant properties of other bakery products such as cakes, buns, cookies, and muffins enriched with apple pomace have been investigated by several researchers. For instance, increased antioxidant properties with improved sensory attributes (fruity flavor) were observed when muffins were prepared with 20% apple pomace [59,60]. Similarly, another study reported that muffins blended with up to 32% apple peel powder showed higher total phenolic content, total antioxidant capacity, dietary fiber content, and water holding capacity [61]. Several published works have investigated the effect of total polyphenol content and total dietary fiber content in cookies enriched with apple pomace (5%, 10%, 15%, 20%, and 25%). The best result was obtained when the cookies were prepared by adding apple pomace powder (10%), with acceptable sensory attributes and nutritional composition [62,63]. Other important applications of apple pomace powder involved the preparation of a gluten-free bread with satisfactory sensory quality and biscuits with a low glycemic index. In detail, biscuit products blended with 10% and 20% dried apple pomace showed decreased glycemic index. It was found that glycemic index values were reduced with increasing concentrations of apple pomace; the values were 65.7 and 60.8 for 10% and 20%, respectively, whereas the value was 70.4 for the control sample. Moreover, the overall results for sensory analysis were quite acceptable, except for the color as it became darker in comparison to the control [64,65]. Finally, the addition of apple and apple pomace powder in bread and bakery products has a positive impact in terms of total phenolic content, total antioxidant capacity, and acceptable sensory properties (except color). Generally, most of the studies reported that bakery products blended with apple pomace powder became darker due to the probability of nonenzymatic browning of apple carbohydrates, which could undergo caramelization or Maillard reaction during heating treatments [63,65].

### 4.2. Fish and Fish Products

Natural antioxidants derived from apples have gained much interest for their antioxidative characteristics. Thus, they have become an important factor in the preservation of food products (containing long-chain unsaturated fatty acids) to prevent lipid oxidation. For example, Rupasinghe et al. [66] applied two different apple peel extracts to protect fish products from oxidation. For this purpose, an initial apple peel extract was prepared using 95% ethanol (including primary and secondary metabolites), whereas the second extract was collected from the first after removing two metabolites (sugars and organic acids). The total phenolic content of the second extract was higher than that of the first, with values equal to 42,025.5 μg/mL and 399.1 μg/mL, respectively. Similarly, the second extract was more potent compared to the first extract in terms of antioxidant capacity (while performing FRAP, ORAC, and DPPH• assays). The antioxidant capacity values for the second extract were 317 (FRAP assay) and 134 (ORAC assay) times higher than the first extract. In addition, lipid oxidation in fish oil was investigated by exposing it to major precursors to initiate oxidation (2,2′-Azobis(2-amidinopropane) dihydrochloride (AAPH), heat, and UV light). The reason for using such types of precursors is to facilitate the irreversible and inevitable oxidative degradation of the double bonds located within unsaturated fatty acids and produce unstable oxidation products. Under these conditions, the inhibition capacity for lipid oxidation of the second extract was comparable to commercial synthetic antioxidants such as butylated hydroxytoluene (BHT). The induction times of BHT (24.5 h with 200 μg/mL) and the second apple extract (25.2 h with 400 μg/mL) resulted higher compared to the induction time (13.6 h) of the first extract (20,000 μg/mL). The study proved that phenolic compounds recovered from apples can act as natural antioxidants depending on their concentration [6,66]. Furthermore, the effect of apple peel extract on lipid and protein oxidation of rainbow trout samples was evaluated during their storage (4 days at 4 °C) by several researchers. For this purpose, trout fish fillets were minced using a meat blender equipped with a 3 mm plate (Pars Khazar Co., Rasht, Iran). Then, three replicates were prepared (80 g of each) for mixing five different concentrations (10, 20, 30, 50, and 100 mg of gallic acid equivalent/kg mince) of apple peel extracts. All samples (control and treated) were kept at 4 °C for 96 h and analyzed at a time interval of 0, 24, 48, and 96 h, respectively.

The formation of secondary (thiobarbituric acid-reactive substances; TBARS) lipid oxidation products was lower in treated trout samples (1.04 mg malonaldehyde (MDA) equivalent/kg) compared to control samples (3.07 mg malonaldehyde (MDA) equivalent/kg) after 4 days of storage. Similar behavior, i.e., lower levels of protein oxidation, was also observed in samples treated with peel extracts [67,68]. Finally, for a fish product (surimi prepared from grass carp fish), the effect of Qinguan young apple polyphenols (especially chlorogenic acid, epicatechin, and phlorizin) on lipid oxidation was tested during storage for 7 days at 4 °C. For this purpose, 0.05% and 0.10% apple polyphenol extracts were added to the surimi samples. The results showed that the formation of lipid oxidation products was lower in comparison to control samples (without antioxidants). The TBARS value was higher (1.3 mg MDA/kg) in the control sample, whereas the sample enriched with apple polyphenol extract showed a lower value (0.3 mg MDA/kg) [69].

### 4.3. Meat and Meat Products

Apple polyphenols obtained from apple pomace and peel could play an important role in inhibiting oxidation (lipid and protein) and retaining the quality of meat and meat products. For instance, Sun et al. [70] evaluated the effect of apple polyphenol concentration ranging from 300 to 1000 ppm on ham products formulated from pork or beef meat. The samples were analyzed in terms of sensory analysis and lipid and protein oxidation during a refrigerated storage period of 35 days. The incorporation of apple peel polyphenols (500 ppm) significantly retained the color of stored pork hams. However, the addition was ineffective for beef hams. All treated samples did not show lipid oxidation, while no significant differences were observed for protein oxidation [70]. Another study was performed to detect total phenolic content and free radical-scavenging activity (RSA) in chicken patty and beef jerky prepared by adding 10% and 20% apple pomace. The results showed that beef jerky with 20% apple pomace had higher RSA in comparison to the control sample [71]. Similarly, chicken sausage blended with 3%, 4%, and 9% apple pomace exhibited an increase in antioxidant activity with higher fiber content and nice color [72].

Lipid and protein oxidation were also analyzed in an industrialized meat product (dry-fried bacon) to observe the effect of apple polyphenol (AP). The TBARS value was lower in bacon treated with AP (300 mg/kg) with a value of 0.59 mg MDA/kg, whereas the control sample presented a value of 1.03 mg MDA/kg. This result indicated that the AP-treated bacon sample delayed the lipid oxidation. Similarly, the addition of the same amount of AP exhibited the strongest inhibitory effect against protein oxidation among all studied plant polyphenols (tea polyphenol and cinnamon polyphenol) that exhibited less effect on the color of the bacon sample. The protein carbonyl value was 2.30 nmol/mg protein (as protein carbonyl content is a useful indicator for the determination of protein oxidation in meat and meat products), whereas the control sample showed a high protein carbonyl content value of 3.21 nmol/mg [72]. Furthermore, the effect of apple pomace on lipid oxidation was evaluated in boneless mutton meat (both cooked and uncooked). For this purpose, 1%, 3%, and 5% apple pomace powder (APP), respectively, was added to mutton meat with 10% fat to prepare goshtaba. The highest inhibitory effect was found for meat samples (both cooked and uncooked) prepared with 5% APP [73].

### 4.4. Functional Packaging Materials

Functional packaging materials, formulated either by adding antioxidants or coating them onto food packaging materials, have gained increasing attention. The purpose of using functional packaging materials is to reduce food oxidation (the main cause of food spoilage) [74]. In addition, several research studies have been performed to develop biodegradable packaging materials incorporated with natural antioxidants for food applications to replace synthetic and non-biodegradable materials [75,76,77]. Functional packaging materials made from polyvinyl alcohol (PVA) matrix incorporated with apple pomace powder (from 1 to 10% *w*/*w*) showed higher antioxidant activity when used as a packaging material to preserve soybean oil stored at 23 or 60 °C. The antioxidant activity was directly proportional to the concentration of apple powder added to PVA. In addition, lipid oxidation of packaging was also evaluated for the same sample in terms of TBARS value. The oxidative index reduced with increasing temperature (23–60 °C). In fact, no differences were observed in TBARS value when samples were stored at 23 °C, whereas it was lower at 60 °C [74]. Another example of a functional packaging material is chitosan film formulated with thinned young apple polyphenols (YAP). It has been reported that chitosan (biopolymer) film with (1% *w*/*v*) YAP exhibited 3 times higher antioxidant activity compared to control film. The reason for the enhanced antioxidant activity was due to the presence of a large amount of polyphenols (chlorogenic acid and phlorizin as major phenolic compounds) in thinned young apple [78]. Chitosan film incorporated with YAP was applied to freshwater fish (grass carp) fillets to evaluate the effects on lipid and protein oxidation during cold storage. The wrapping of the fish fillet with chitosan–YAP film allowed the protection of the product from both lipid and protein oxidation. The results showed that the formation of peroxides was lower in the wrapped fillet in comparison to the control and thus the lipid oxidation process was retarded. Similar results were observed in the wrapped fillet for protein oxidation as the formation of total volatile basic nitrogen was decreased. The inhibitory activity of the film against lipid and protein oxidation was considered to be due to the antioxidant properties of thinned young apple [77]. The effect of apple by-product (peel and pomace) polyphenols incorporated into chitosan (CS) film was also investigated in several studies. For CS-based apple peel polyphenol (1% *w*/*v*) film, enhanced antioxidant and antimicrobial activities were observed in comparison to the control film. The radical-scavenging activity was increased nearly 4-fold for both DPPH and ABTS assays, and the antimicrobial activity against *Bacillus cereus, Escherichia coli, Salmonella typhimurium*, and *Staphylococcus aureus* was increased more than 2-fold [79]. The effect of Fuji apple polyphenol as a coating material (apple peel powder and carboxymethylcellulose) was also evaluated in patties prepared from beef during 10 days of storage in the refrigerator. The use of the active coating was able to protect uncooked beef patties from lipid oxidation and microbial growth without changing the sensory attributes [80].

Furthermore, CS-based film with 5% *w*/*v* red apple pomace extract (APE) exhibited the strongest antioxidant properties, while CS-based film with nanosized TiO_2_ and APE showed the highest antimicrobial activity among all studied films (CS film; CS-APE film; CS-TiO_2_ film; CS-TiO_2_-APE film). The radical-scavenging ability in a DPPH assay was increased by nearly 4 times, and the antimicrobial activity against Escherichia coli and Staphylococcus aureus was increased by 7 and 8 times, respectively [75]. 

Finally, the antioxidant activity and lipid oxidation of edible films prepared with cassava starch, sodium carboxymethyl cellulose (CMC), and apple polyphenol (AP) were evaluated. Results obtained from DPPH and ABTS radical-scavenging activity assays showed increased antioxidant activity in comparison to control film, and a dose-dependent response was observed (based on the concentration of AP added in the films) for antioxidant activity. The results of a lipid oxidation assay for chicken meat during storage at 4 °C for 7 days exhibited a lower value for TBARS. The value was 0.092 mg MDA/kg (which was much lower than the control film with a value of 0.178 mg MDA/kg). The optimum concentration of AP was 70 mg/mL for this edible film, based on its oxidation inhibition capacity as well as other physical and mechanical properties [76]. It can be concluded that the addition of apple polyphenols in packaging materials (either natural or synthetic) showed a better inhibitory effect against lipid and protein oxidation and better antioxidant performance. Therefore, packaging materials incorporated with apple polyphenols could be a potential alternative to ensure food safety and shelf-life extension for highly perishable food items (fish, meat, fruit, and vegetables).

### 4.5. Other Products

The antioxidant properties of other food products such as noodles, vegetable juices, and yoghurt were also investigated when apple pomace was added. In detail, noodles were formulated with 10% and 25% apple pomace. The incorporation of 10% apple pomace powder in samples had a positive effect on nutritional properties in terms of dietary fiber content, protein content, and antioxidant activity. Also, the sensory attributes (color, flavor, taste, and texture) were improved without affecting the cooking or texture properties of noodles. On the other hand, noodles produced with 25% apple pomace in the wheat flour were significantly affected in terms of color, flavor, taste, and texture [81].

Similarly, a study on carrot and tomato juices revealed that both samples enriched with apple peel extracts showed an increase in the antioxidant capacity, and the increased value was equal to 160 mg gallic acid equivalent (GAE)/L. For this purpose, the oxidative stability of juices enriched with apple peel extracts and commercial antioxidants (BHT/BHA) was evaluated. The samples treated with apple peel extracts (20 mg/L of GAE) and commercial antioxidants (25 μM) were stable during their storage, while for the control samples, high lipid hydroperoxides were reported with values above the threshold [82].

Another important potential application of apple extracts is the fortification of yoghurt. The use of apple pomace extracts in yogurt formulation provided a final product with improved fiber content and antioxidant properties compared to plain yogurt. For instance, yoghurt fortified with apple pomace extracts (3.3%) showed an enhancement of the total phenolic content and antioxidant activity. The values were 2 times and 3 times higher, respectively, in comparison to the control [83]. The effect of apple peel extract (APE) on probiotic yoghurt was also investigated. In this regard, probiotic yoghurt was made by adding different concentrations of APE (1%, 2%, 3%, 4%, and 5%, respectively). The results exhibited higher total phenolic content and antioxidant capacity in fortified probiotic yoghurts compared to the control sample. The enhancement of total phenolic content and antioxidant capacity was correlated to the quantity of APPE added to probiotic yoghurts. Therefore, probiotic yoghurt formulated with 5% APE showed the highest total phenolic content and inhibition of oxidation (expressed as a percentage) among all with values equal to 9.52 g GAE/100 g of DW and 47%, respectively, during 21 days of refrigerated storage [84].

**Table 1:** Application of apple polyphenols in food products.

	Food Product	Formulation	Apple Variety	Outcomes	References
Bakery	Bread	Incorporation of defatted apple seed powder (5% and 20%)	Golden Delicious, Idared, and Šumatovka	Incorporation of 5% apple seed powder: TPC and TAC increased 1.7- and 1.1-fold Incorporation of 20% apple seed powder: TPC and TAC increased 2.9- and 2.1-fold	[58]
	Gluten-free bread	Addition of apple pomace (up to 12.5%)	-	Satisfactory sensory quality TPC increased TAC increased 5.8-fold (ORAC)	[64]
	Muffins	Addition of apple peel powder (up to 32%)	Idared and Northern Spy	TAC increased 4.9-fold (FRAP)	[61]
	Cookies	Addition of apple pomace powder (5%, 10%, 15%, 20%, and 25%)	-	TPC increased	[62]
	Biscuits	Addition of apple pomace (10% and 20%)	Golden Delicious	TPC increase and reduced glycemic index (65 and 60, respectively)	[65]
Fish and fish products	Rainbow trout fish	Apple peel extracts (from 10 to 100 mg/kg of fish)	-	TPC and TAC increased	[67]
	Surimi	Young apple extracts	Qinguan	Lipid and protein oxidation retarded	[69]
	Fish oil	Apple peel extracts (400 µg/mL of fish oil)	Northern Spy	TPC and TAC increased (ORAC and FRAP); lipid oxidation retarded	[66]
Meat and meat products	Ham	Apple extracts (300 mg/kg)	-	Lipid oxidation retarded	[70]
	Beef jerky	Wet apple pomace (10 and 20%)	-	TPC and TAC increased	[63]
	Chicken sausages	Apple pomace (3%, 4%, and 9%)	-	TAC increased	[71]
	Bacon	Apple polyphenol powder (300 mg/kg)	-	Lipid and protein oxidation retarded	[72]
	Mutton	Apple pomace powder (1%, 3%, and 5%)	-	Lipid oxidation retarded	[73]
	Polyvinyl alcohol matrix	Apple pomace powder (1%, 5%, and 10%)	-	TPC and RSA increased; Lipid oxidation retarded	[74]
	Chitosan film	Young apple polyphenols (1% *w*/*v*)	-	RSA increased; Lipid and protein oxidation retarded	[77,78]
Functional packaging materials	Edible coating material	Apple peel powder	Fuji	Lipid oxidation and microbial growth retarded	[80]
	Edible film	Apple polyphenols powder (from 40 to 80 mg/mL)	-	RSA increased; Lipid oxidation retarded	[76]
Others	Juices (Carrot and tomato)	Apple peel extracts (from 40 to 800 mg/L)	Bramley	TAC increased	[82]
	Yoghurt	Apple pomace extracts (3.3%)	Royal Gala	TPC and TAC increased	[83]
	Probiotic yoghurt	Apple peel extracts (1–5%)	Red Delicious Golden Royal Gala	TPC and antioxidant capacity increased	[84]

TPC: total phenolic compounds; TAC: total antioxidant capacity; ORAC: oxygen radical absorption capacity; FRAP: ferric reducing antioxidant power; RSA: radical-scavenging activity.

## 5. Conclusions

Apples and apple by-products are rich sources of phenolic compounds. Therefore, the extraction of phenolic compounds from apples and their application in processed foods as natural food additives could play an important role in replacing synthetic antioxidants and conventional packaging materials. The high phenolic content of apples and apple by-products (powder and extracts) facilitates the development of final products with increased antioxidant properties without affecting the sensory attributes. In addition, the proper utilization of apples and apple by-products minimizes the detrimental effect on the environment and contributes to a positive effect on the economy. However, when moving to the application, the main drawback found for fortified bakery products is the undesirable change in color (darker and brownish). On the other hand, the alteration of color in meat products was assessed positively by sensory panelists. This underlines the need to perform more studies to investigate the effect of the addition of such ingredients to foods.

Moreover, more investigations are also required regarding safety issues of apple-derived products, especially for apple by-products as they contain measurable levels of pesticide residues. They can be effectively removed by applying some techniques such as ozonation and washing (with sodium bicarbonate) prior to processing. Other concerns regarding safety issues of apple-derived products include the release of cyanide glycosides from apple seeds and the accumulation of patulin due to fungal growth (its maximum level has been established by the European Food Safety Authority (EFSA) and the United States Food and Drug Administration (FDA) as equal to 50 μg/kg (of product) for adults and 10 μg/kg for infants and young children).

These conclusions clearly emphasize the need to take into account several issues for future applications dealing with apples and apple-derived by-products.
